# Succinic acid improves crop chemical components by regulating soil chemical properties, microbes and metabolites

**DOI:** 10.3389/fmicb.2025.1674309

**Published:** 2025-10-21

**Authors:** Dian-long Li, Qiang Liu, Rui-juan Zhao, Wei-chang Gao, Xin-rong Zheng, Shi-yu Zhou, Kai Cai, Xian-jun Jiang

**Affiliations:** ^1^College of Resources and Environment, Southwest University, Chongqing, China; ^2^Upland Flue-Cured Tobacco Quality & Ecology Key Laboratory of CNTC, Guizhou Academy of Tobacco Science, Guiyang, Guizhou, China; ^3^Qiandongnan Tobacco Company of Guizhou Province, Kaili, Guizhou, China

**Keywords:** succinic acid, microbial community, soil metabolomics, correlation analysis, tobacco chemical components

## Abstract

**Introduction:**

Small molecule metabolites can act as soil conditioners to improve the soil environment and thereby promote crop growth. Like many other components of root exudates, succinic acid not only contributes to plant growth and stress resistance but also influences microbial growth in soil, thereby participating in the carbon cycle. Succinic acid is believed to act as a signaling molecule that bridges the host plant and microorganisms during their interactions. However, the mechanism by which succinic acid affects microbes, metabolites and their interaction in soil remains unclear.

**Methods:**

High-throughput sequencing and pseudotargeted metabolomics techniques were applied for exploring the effects of succinic acid in tobacco-planting soil and corresponding tobacco chemical composition.

**Results:**

The addition of succinic acid improved the soil chemical properties for increasing the available potassium, total nitrogen, total phosphorus and total potassium, and had a positive impact on soil fertility. In the microbial communities, fungi were more sensitive to succinic acid than bacteria. The relative abundance of *Proteobacteria* in the bacterial community was significantly decreased, while that of *Chloroflexi* was significantly increased. The relative abundances of *Actinobacteriota* and *Acidobacteriota* also showed a decreasing trend. The relative abundances of *Ascomycota* and *Basidiomycota* in the fungal community increased significantly, while the relative abundance of *Chytridiomycota* decreased significantly. The microbial function prediction indicated that 0.4% succinic acid may affected the nutrients and carbon-nitrogen cycles. In the soil metabolomics, the absolute contents of monosaccharide, disaccharide, sugar alcohol and trehalose in soil metabolites increased significantly. The 47 characteristic metabolites were significantly enriched in amino acid metabolism and carbohydrate metabolism. Correlation analysis showed that soil microbes were mainly positively correlated with amino acids and sugars. In addition, the relative abundances of monosaccharide, disaccharide and sugar alcohol increased in tobacco leaf, while alkaloid and amino acid decreased for improving the tobacco chemical composition.

**Conclusion:**

This study demonstrated that the addition of succinic acid affected soil chemical property, microbial communities and the composition of soil metabolites, and then improved crop chemical components.

## Graphical Abstract

**Figure:**
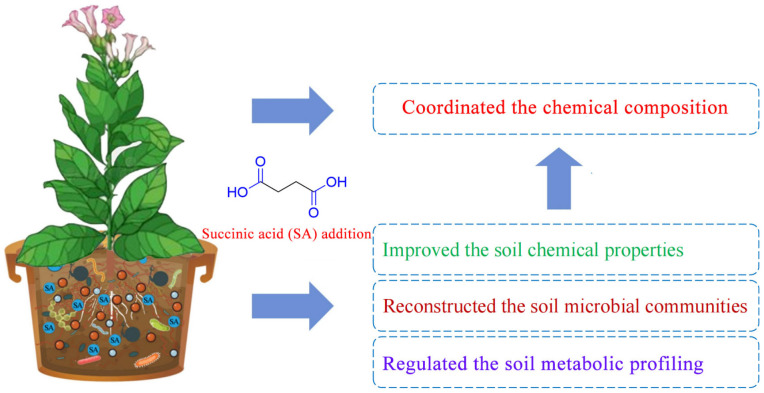
Exogenous succinic acid application in soil regulates the chemical composition of tobacco leaves.

## 1 Introduction

Tobacco is one of the important cash crops in China and tobacco leaf can be used in the cigarette, medical and biofuel industries (Gao et al., 2019). The soil ecological environment determines the yield, quality and aroma characteristics of tobacco (Yan et al., 2022). However, at present, agricultural production practices generally faces problems such as the difficulty in improving yield and quality, the aggravation of soil-borne diseases, and the serious obstacles to continuous cropping (Rajesaheb et al., 2025). In order to improve soil condition and ensure the crop quality, an effective soil improvement strategy is urgently needed for promoting the healthy development of agriculture.

Soil microbes, as important participants in soil activities, can promote the decomposition of soil organic matter, accelerate the transformation and circulation of soil nutrient elements, and store effective nutrients for plants. At the same time, they can respond quickly to environmental changes and are an important indicator of soil ecological environment quality (Srikanth et al., 2025). Manipulating the plant holobiont through microbiome engineering to enhance crop yield quality and resistance to environmental stress is an emerging biotechnology strategy. Inoculating or recruiting a combination of beneficial microbes that can promote the healthy growth of crops to alter the microbiome can promote crop growth and development and alleviate pathogen and abiotic stress (Arif et al., 2020). Recently, soil improvement using low-molecular-weight organic compounds (such as root secretions and secondary metabolites produced by soil microbes) as soil conditioners has become a research hotspot in the agricultural field (Fallah et al., 2023). These metabolites can enhance soil physicochemical properties, increase enzyme activity, enrich beneficial metabolites, and regulate the structure and function of microbial communities.

The composition of soil metabolites includes secretions from plant roots, metabolic decomposition of organic matter by microbes, and endogenous metabolites of microbes themselves (Brown et al., 2024). Common categories include fatty acids, sugar acids, sugar alcohols, sugars, amino acids, polyols, purines, and other organic metabolites. Their distribution strongly affects the soil physicochemical properties, the availability of nutrients, the structural composition and dynamic changes of the microbiome (Koegel-Knabner and Rumpel, 2018; Withers et al., 2020), which provides the possibility of using these low-molecular-weight metabolites to purposefully regulate the microbiome and improve the ecological environment (Kallenbach et al., 2016). The complex soil microbial community is a key factor for maintaining the healthy growth of plants and the stability of the ecological environment (Yu et al., 2022; Bolan et al., 2011). When plants are subjected to environmental stress or attacked by various disease-causing microbial pathogens during their growth process, the application of low-molecular-weight metabolites can recruit specific beneficial microbes to enhance their ability to resist adverse conditions. For example, *N*-acetylglucosamine can promote the growth of tomato plants by shaping the community structure and metabolism of the rhizosphere microbiome (Sun et al., 2022). Acetic acid alters the microbes and metabolic composition in the rhizosphere to enhance the drought resistance of willow trees (Kong et al., 2022). The application of a mixture of long-chain fatty acids and amino acids can induce a pathogen inhibition response mediated by the soil microbiome, thereby enabling *Arabidopsis thaliana* to develop resistance to inhibit pathogen infection in leaves (Yuan et al., 2018). Therefore, some small molecule soil metabolites have been identified as plant signals involved in shaping the plant-soil microbiome (Huang et al., 2019).

Small molecule metabolites can be directly used as carbon sources by soil microbes. The metabolism of these organic compounds by soil microbes in turn affects the soil microbial community and the functions of plant roots. Therefore, there is a significant natural interaction between soil microbes and small molecule metabolites (Ren et al., 2022). This phenomenon can promote the interaction among soil metabolites, crops and the microbial community. This affects the health of the soil and the growth and development of crops, opening up a broad field for the application of small molecule metabolites in agricultural practice. Succinic acid is an organic acid component of root exudates, serving as an important regulator of plant growth and a key modulator of the rhizosphere microbiome. Succinic acid functions both as a carbon source and a signaling molecule, significantly influencing the structural and functional dynamics of the rhizosphere microbiome, thereby promoting plant health (Wang et al., 2025; Song et al., 2018). Evidence suggests that succinic acid can enhance the growth and activity of beneficial microbial populations while inhibit the proliferation of pathogenic microorganisms (Wu et al., 2011). Additionally, succinic acid induces changes in soil microbial metabolism, leading to the biosynthesis of metabolites that enhance the plant’s disease resistance (Swenson et al., 2015). The functions and roles of some other small molecule metabolites in soil have been explored and developed (Sun et al., 2022; Kong et al., 2022; Yuan et al., 2018; Ström et al., 2005; Reischke et al., 2014; Wu et al., 2020), but there are few reports on the application of succinic acid for the interaction among soil metabolites, crops and the microbial community. Therefore, different concentrations of succinic acid were applied for studying the effect on tobacco-planting soil. The soil chemical properties, microbial communities and functions, metabolites and tobacco leaf metabolites were analyzed with high-throughput sequencing and pseudotargeted metabolomics to clarify their regulatory mechanisms. This study aims to: (1) explore the effects of different concentrations of succinic acid on the chemical properties, microbes and metabolites in tobacco-planting soil; (2) reveal the interaction between the microbial community and metabolites; (3) clarify the mechanism of succinic acid effect on the ecological environment; (4) analyze the influence of the synergistic regulation of soil microbial communities and metabolites on the crop chemical components. This research is conducive to a comprehensive understanding of the action mechanism of the small molecule soil metabolite of succinic acid.

## 2 Materials and methods

### 2.1 Experimental sites

The experimental site is located in the southwest plateau area of China, Guanshanhu District (25°45′∼26°31′N, 106°25′∼106°41′E), Guiyang City, Guizhou Province. This area has a north-subtropical humid and mild climate, with an altitude about 1,200 m. The mean annual temperature, relative humidity, sunshine duration and precipitation are 15.3 °C, 77%, 1148.3 h and 1129.5 mm, respectively. The yellow clay-loam soil is the most widely distributed tobacco-planting soil, which was used in the pot experiment. The soil sample was obtained from the surface layer (0–20 cm) in tobacco base of Pingba District, Anshun City, Guizhou Province (26°26′ N, 106°14′ E).

### 2.2 Experimental design

According to the literature (Ström et al., 2005), the addition of the small molecular organic acid citric acid to soil can affect rhizosphere nutrient cycling, with a medium concentration of 0.48%. Therefore, this experiment design was set up with four treatments: T0, normal fertilization (CK); T1, addition of 0.1% succinic acid (LSA); T2, addition of 0.2% succinic acid (MSA); T3, addition of 0.4% succinic acid (HSA), and each treatment was performed with 5 replications with blocking randomization method. The small molecule metabolite succinic acid (CAS: 110-15-6, 99.5%) was purchased from Shanghai Maclin Biochemical Technology Co., Ltd., Plastic pots with a height of 46 cm, a diameter of 50 cm and a bottom diameter of 32 cm were selected. First, 5 kg sifted soil was weighed and mixed with small-molecule metabolites evenly. The soil was placed in pots and the special compound fertilizer for tobacco (N:P_2_O_5_:K_2_O = 10:10:25) was added. Second, the water was added in the bottom of potted plant to saturate the soil. Finally, the Yunyan 87 tobacco was transplanted into potted soils with the same growth condition at the large cross stage. The soil and tobacco samples were collected after planting at 90 days.

### 2.3 Sample collection

The near-rhizosphere soil samples were collected from each treatment, after removing the fine root and other impurities. These were divided into three parts for analysis. The first part was naturally air-dried, ground, and then stored after passing through a 3 mm sieve for determination of the chemical properties. The second part was stored with Rnase/DNase-free centrifuge tube in dry ice and analyzed for microbial amplification sequencing. 16S rRNA and ITS gene sequencing analyses were conducted on soil bacteria and fungi, respectively. The third part was stored in liquid nitrogen, freeze-dried in a vacuum freeze dryer, and kept at −80 °C for soil metabolite analysis. Meanwhile, the 6–7 leaves (from bottom to top in) were collected with the half-leaf method and stored in liquid nitrogen. After being freeze-dried by a vacuum freeze dryer, they were stored at −80 °C for the analysis tobacco chemical components.

### 2.4 Soil chemical property analysis

The pH of the soil solution was determined by a pH meter, and the ratio of soil to aqueous solution (w/v) was 1:2.5. The soil total nitrogen (TN) was determined using a continuous flow analyzer based on the potassium dichromate-sulfuric acid digestion method. The soil alkali-hydrolyzed nitrogen (AHN) was determined using a Kjeldahl nitrogen analyzer based on the wet alkali digestion method. The total phosphorus (TP) and total potassium (TK) were determined by sodium hydroxide melting-molybdenum antimony anti-colorimetric method and sodium hydroxide melting-atomic absorption spectrophotometry. Available phosphorus (AP) and available potassium (AK) were extracted with 0.5 M NaHCO_3_ (pH 8.5) at a ratio of 1:20 (w/v) and 1 M CH_3_COONH_4_ (pH 7.0) at a ratio of 1:10 (w/v), respectively, and then determined via a continuous flow analyzer and atomic absorption spectrometry. The soil organic matter (SOM) was extracted with 0.4 M potassium dichromate and a sulfuric acid solution at a ratio of 1:100 (w/v) and determined via the titration method (Bao, 2000).

### 2.5 Soil DNA extraction and sequencing

The genomic DNA of the samples was extracted via CTAB, and then the DNA purity and concentration were determined via agarose gel electrophoresis. Using the diluted genomic DNA as a template, PCR was performed via specific primers with Barcode and Phusion^<reg>(</reg>^ High-Fidelity PCR Master Mix with GC Buffer (New England Biolabs, China). The primers 515F (5′-GTGCCAGCMGCCGCGGTAA-3′) and 806R (5′-GGACTACHVGGGTWTCTAAT-3′) were used to amplify the V3−V4 region of the bacterial 16S rRNA gene to characterize bacterial diversity. The primers ITS5-1737F (5′-GGAAGTAAAAGTCGTAACAAGG-3′) and ITS2-2043R (5′-GCTGCGTTCTTCATCGATGC-3′) were used to amplify the internal transcribed spacer region 1 (ITS1) of the fungi to characterize fungal diversity.

### 2.6 Soil environmental pseudotargeted metabolomics analysis

Environmental pseudotargeted metabolomics was applied for soil metabolite analysis. A total of 1.5, 0.8 and 0.8 ml of methanol: water (v/v = 3:2) were added in 0.5 g soil for sequentially vortex extracting soil metabolites at 4 °C. The extract solution was combined, freeze-dried and then derivatized. A total of 20 μl of methoxyamine hydrochloride (25 mg ml^1^) and 100 μl of N,O-Bis(trimethylsily)trifluoroamide (1% chlorotrimethylsilane) was used for oximation and silylation. The reaction was carried out (3 + 2 min) and (3 + 3 + 3 min) conditions with 800 W of microwave energy. The supernatant was analyzed via GC–MS with a 1 μl injection. The identification and quantification parameters are described in the literature (Cai et al., 2023b).

### 2.7 Tobacco pseudotargeted metabolomics analysis

Tobacco pseudotargeted metabolomics was applied for tobacco chemical components. 3 ml of methanol: CHCl_3_: water (v/v/v = 2.5:1:1) was added in 50 mg leaf powder for ultrasonic extracting metabolites of tobacco leaf. After centrifugation, 300 μl supernatant was dried with N_2_ flow at room temperature and then further dried completely by adding 300 μl of dichloromethane. A total of 40 μl of methoxyamine hydrochloride (30 mg mL^1^) was used for oximation. The reaction was carried out in a constant temperature at 41 °C for 120 min. And then, 200 μl of N,O-Bis(trimethylsily)trifluoroamide (1% chlorotrimethylsilane) was added for silylation. The reaction was carried out in a constant temperature at 81 °C for 90 min. The supernatant was analyzed via GC–MS with a 1 μl injection. The identification and quantification parameters are described in the literature (Jing et al., 2024).

### 2.8 Statistical analysis

The soil physicochemical was statistically analyzed using One-way analysis of variance (ANOVA) with Duncan’s post-hoc test in IBM SPSS Statistics to determine whether concentration levels were significantly different between control and succinic acid treatments (*p* < 0.05 was regarded as significance and the results were shown as mean ± standard deviation). Alpha diversity indices Chao1 and Shannon index were calculated in QIIME. Principal coordinate analysis (PCoA) was done and visualized in R with packages WGCNA using function hclust and ggplot2. Microbial venn diagrams, bar charts, heat maps, and the cumulative bar charts and percentage bar charts of soil metabolites and tobacco leaf metabolites were drawn using Origin 2018. Principal component analysis, screening of characteristic metabolites (*p* < 0.05, VIP > 1.2) and metabolic pathways analysis of soil metabolites were conducted using MetaboAnalyst 6.0.^1^ The Pearson’s correlation analysis (r > 0.6 or r < −0.6, *p* < 0.05) of characteristic metabolites and differential microbes were analyzed and drawn with R V2.15.3 using packages corrplot, ggplot2 and ggpubr.

## 3 Results

### 3.1 Soil chemical properties

The analysis of soil chemical properties showed that the addition of succinic acid significantly decreased the soil pH, and increased the contents of AK, TN, TP, and TK, especially for higher contents of AHN and OM in 0.4% succinic acid. Compared with the CK, the addition of 0.4% succinic acid effectively regulated the soil chemical properties and had a positive effect on soil fertility (Table 1).

**TABLE 1:** Changes in chemical properties in the tobacco-planting soil.

Treatment	pH (KCl)	AHN (mg kg^1^)	AP (mg kg^1^)	AK (mg kg^1^)	TN (%)	TP (%)	TK (%)	OM (g kg^1^)
CK	5.92 ± 0.17a	141.30 ± 16.44a	110.68 ± 18.96a	1107.60 ± 76.99b	0.192 ± 0.007b	0.119 ± 0.032a	1.43 ± 0.03b	32.32 ± 0.65a
LSA	5.60 ± 0.09b	146.74 ± 8.92a	105.38 ± 14.40a	1190.40 ± 22.32ab	0.199 ± 0.005b	0.171 ± 0.015a	1.44 ± 0.02b	30.92 ± 1.75a
MSA	5.56 ± 0.05b	140.14 ± 5.34a	125.14 ± 13.16a	1221.60 ± 68.47a	0.201 ± 0.011b	0.134 ± 0.072a	1.51 ± 0.04a	31.30 ± 1.26a
HSA	5.68 ± 0.15b	149.60 ± 13.84a	111.06 ± 14.44a	1267.20 ± 71.44a	0.216 ± 0.006a	0.170 ± 0.024a	1.52 ± 0.02a	35.50 ± 1.09a

ANH, alkali hydrolyzed nitrogen; AP, available phosphorus; AK, available potassium; TN, total nitrogen; TP, total phosphorus; TK, total potassium; OM, organic matter. Different lowercase letters in the same column indicate that significant differences between treatments (*p* < 0.05).

### 3.2 Soil microbial community

The bacterial and fungal communities were cluster analyzed at 97% similarity level, and the Venn graph was drawn (Supplementary Figure 1). Out of the bacterial OTUs, 4,403 were detected in CK, 4,070 in LSA, 3,925 in MSA, 4,284 in HSA, 1,140 in all treatments. Out of the fungal OTUs, 2,176 were detected in CK, 1,474 in LSA, 1,300 in MSA, 1,071 in LSA, 318 in all treatments. The addition of succinic acid decreased the total OTUs of bacteria and fungi, and the decrease was more pronounced in fungi than in bacteria. Meanwhile, it also significantly decreased the alpha diversity index of bacterial and fungal communities (Supplementary Figure 2).

**FIGURE 1:**
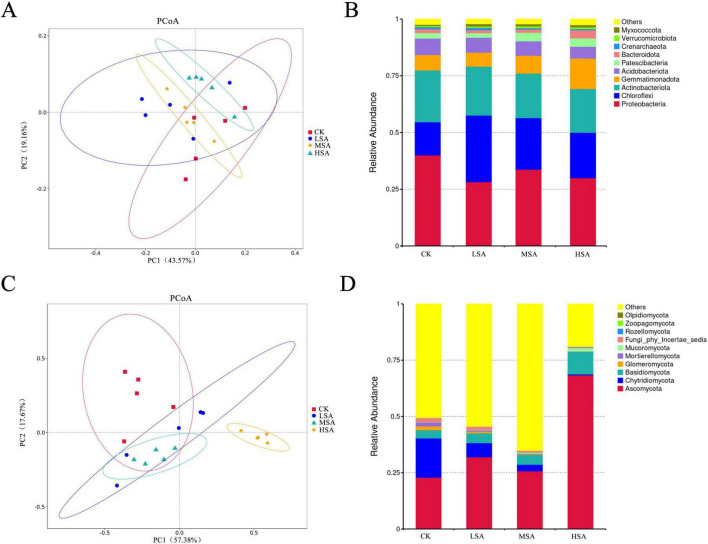
Principal co-ordinates analysis (PCoA) of soil bacterial **(A)** and fungal **(C)** communities in the tobacco-planting soil on the basis of weighted unifrac treated with different concentrations of succinic acid (CK, normal fertilization; LSA, 0.1% succinic acid; MSA, 0.2% succinic acid; HAS, 0.4% succinic acid). Relative abundance histograms of the bacterial **(B)** and fungal **(D)** community structures at the phylum level.

In order to further explore the changes of microbial communities with different concentrations of succinic acid, PCoA analysis was conducted based on Weighted unifrac distance, and the first two components of bacterial community accounted for 54.36% of the variation, while the first two components of fungal community accounted for 71.38% of the variation. Compared with the bacterial community, the fungal community had higher explanatory power and was more sensitive to the addition of succinic acid (Figures 1A, C). Compared with CK, the HSA shifted significantly, indicating that the addition of high concentration of succinic acid regulated the fungal community more obvious.

Changes in bacterial community structure at the phylum level showed that the addition of succinic acid inhibited the relative abundance of Proteobacteria and increased the relative abundance of Chloroflexi. The relative abundance of Actinobacteriota and Acidobacteriota has also decreased. The addition of high concentration succinic acid increased the relative abundance of Gemmatimonadota, from 6.82% to 13.46%. In the fungal community, the relative abundance of Ascomycota and Basidiomycota increased, while the relative abundance of Chytridiomycota decreased. Among the three treatments, the effect of high concentration succinic acid was the most significant. The relative abundance of Ascomycota increased from 22.86% to 68.30%, and that of Chytridiomycota decreased from 17.45% to 0.45%. Moreover, the relative abundance of Basidiomycota increased from 3.65% to 9.97%. The addition of succinic acid to tobacco-planting soil had an important effect on both bacterial and fungal communities at phylum level (Figures 1B, D). Further, the changes in the bacterial community at the genus level (Supplementary Figure 3A) indicated that the addition of succinic acid reduced the abundance of Dyella, Rhodanobacter, Chujaibacter, and Castellaniella in the Proteobacteria phylum. The addition of high-concentration succinic acid increased the abundances of Massilia, Sphingomonas, Azoarcus and Ramlibacter in the Proteobacteria phylum. In the fungal community (Supplementary Figure 3B), the addition of succinic acid reduced the abundance of fungal genera such as Boothiomyces, Sagenomella, Humicola, and Paraglomus. Low concentration of succinic acid increased the abundance of Cladorrhinum, Fusicolla, Setophoma, and Preussia in the Ascomycota phylum. High concentration of succinic acid increased the abundance of Penicillium, Trichoderma, and Purpureocillium in the Ascomycota phylum.

**FIGURE 2:**
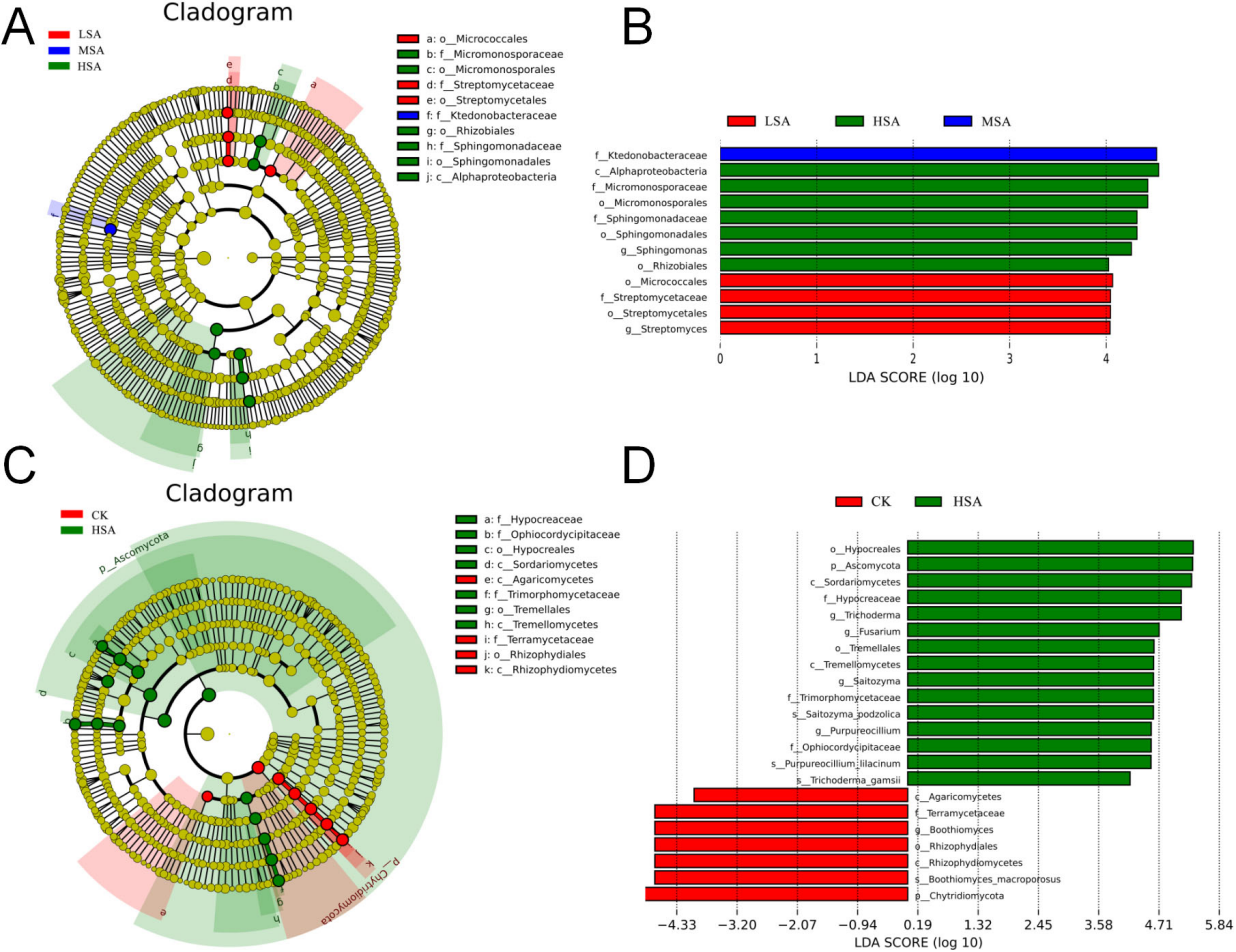
Key bacterial **(A)** and fungal **(C)** communities in tobacco-planting soil response to different concentrations of succinic acid obtained via LEfSe analysis (significantly discriminant taxon nodes are colored and branch areas are shaded according to the highest-ranked variety for that taxon. For each taxon detected, the corresponding node in the taxonomic cladogram is colored by their corresponding group color. If the taxon representation is not significantly different between sample groups, the corresponding node is colored yellow). A total of 12 differentially bacterial abundant taxonomic clades **(B)** and 22 differentially fungal abundant taxonomic clades **(D)** with an LDA score higher than 4.0.

The taxonomic cladogram of microbial communities after addition of different concentrations of succinic was shown in the Figure 2. Significant bacterial biomarkers mainly include *Micrococcales*, *Streptomycetaceae*, *Streptomycetales* in LSA and *Micromonosporaceae*, *Micromonosporales*, *Rhizobiales*, *Sphingomonadaceae*, and *Sphingomonadales* in HSA. In the fungal community, the abundance of fungal communities such as *Chytridiomycota*, *Rhizophydiomycetes*, *Boothiomyces* were significantly inhibited, however, the abundances of fungal communities such as *Hypocreales*, *Sordariomycetes*, and *Trichoderma* were promoted.

The FAPROTAX functional prediction of bacteria (Supplementary Figure 4A) showed that the addition of succinic acid inhibited manganese oxidation, aromatic compound degradation, phototrophy, and enhanced predatory or exoparasitic. Meanwhile, high concentrations of succinic acid significantly enhanced nitrate reduction, nitrogen respiration, nitrate respiration, chemoheterotrophy, and aerobic chemoheterotrophy. The FUNGUILD functional prediction of fungi (Supplementary Figure 4B) showed that the addition of succinic acid inhibited arbuscular mycorrhizal, dung saprotrop−wood saprotroph, dung saprotroph-undefined saprotroph-wood saprotroph, wood saprotroph. Meanwhile, high concentrations of succinic acid significantly enhanced plant pathogen-undefined saprotroph, fungal parasite-undefined saprotroph, fungal parasite, plant pathogen-soil saprotroph-wood saprotroph, endophyte-plant pathogen.

### 3.3 Soil metabolites

**FIGURE 3:**
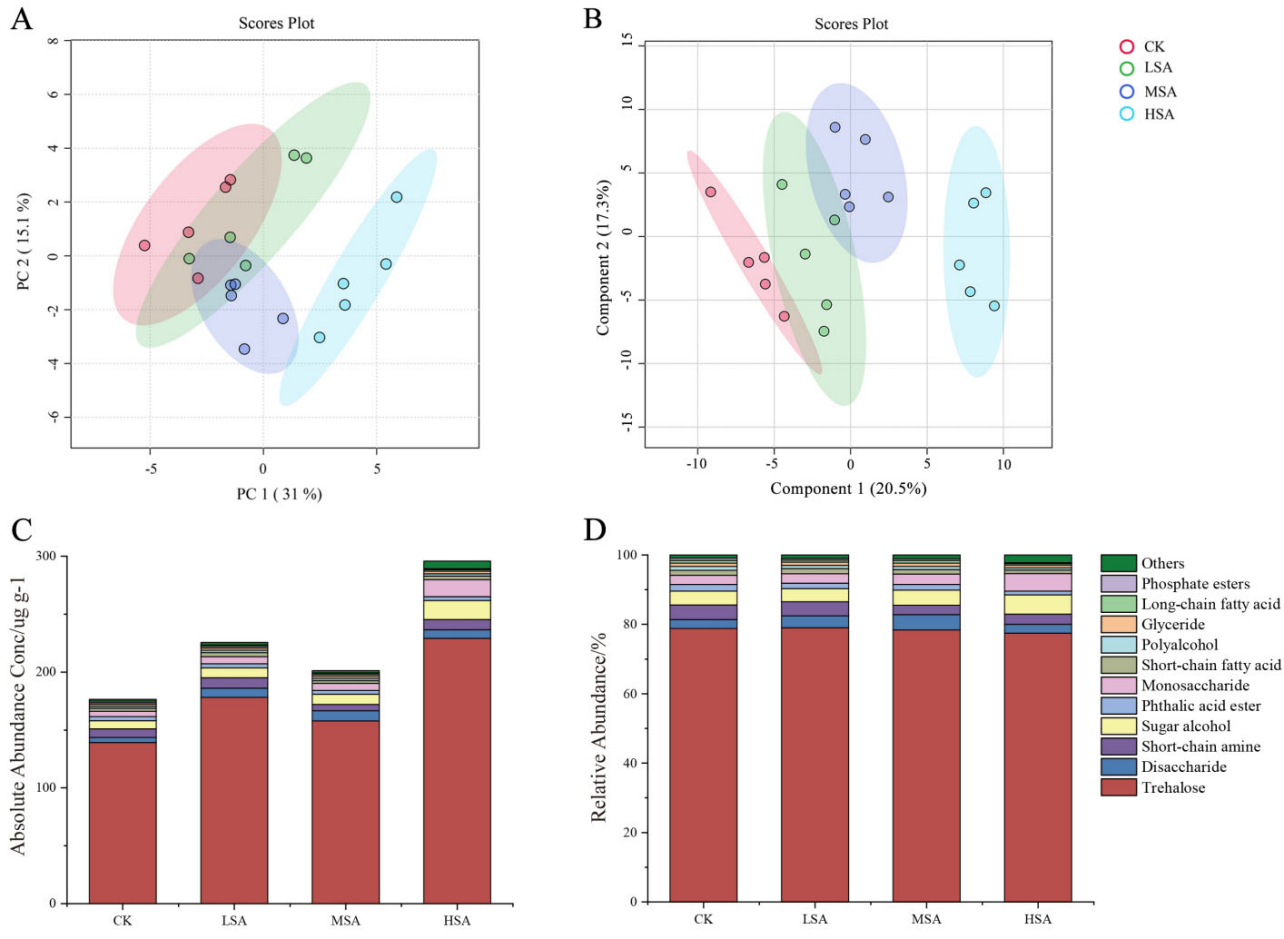
The principal component analysis figure **(A)** and partial least squares discriminant analysis figure **(B)** of soil metabolites treated with different concentrations of succinic acid. The absolute abundance **(C)** and relative abundance **(D)** of the top 11 and other chemical classifications of tobacco-planting soil metabolites treated with different concentrations of succinic acid (CK, LSA, MSA, and HSA).

Both PCA and PLS-DA of the soil metabolites revealed that MSA and HSA were significantly different from the control group, indicating that succinic acid played a certain regulatory role in the composition of soil metabolites (Figures 3A, B). After addition of succinic acid, the total contents of soil metabolites significantly increased. The contents of different types of soil metabolites also changed significantly, 0.1% succinic acid increased the contents of trehalose, disaccharide, short-chain amine, sugar alcohol, monosaccharide and short-chain fatty acid. A total of 0.2% succinic acid increased the contents of trehalose, disaccharide, sugar alcohol and monosaccharide. A total of 0.4% succinic acid significantly increased the contents of trehalose, disaccharide, sugar alcohol and monosaccharide (Figure 3C). Although the absolute content of metabolites in tobacco-planting soil had changed significantly, the relative content of different types of metabolites changed relatively small (Figure 3D).

**FIGURE 4:**
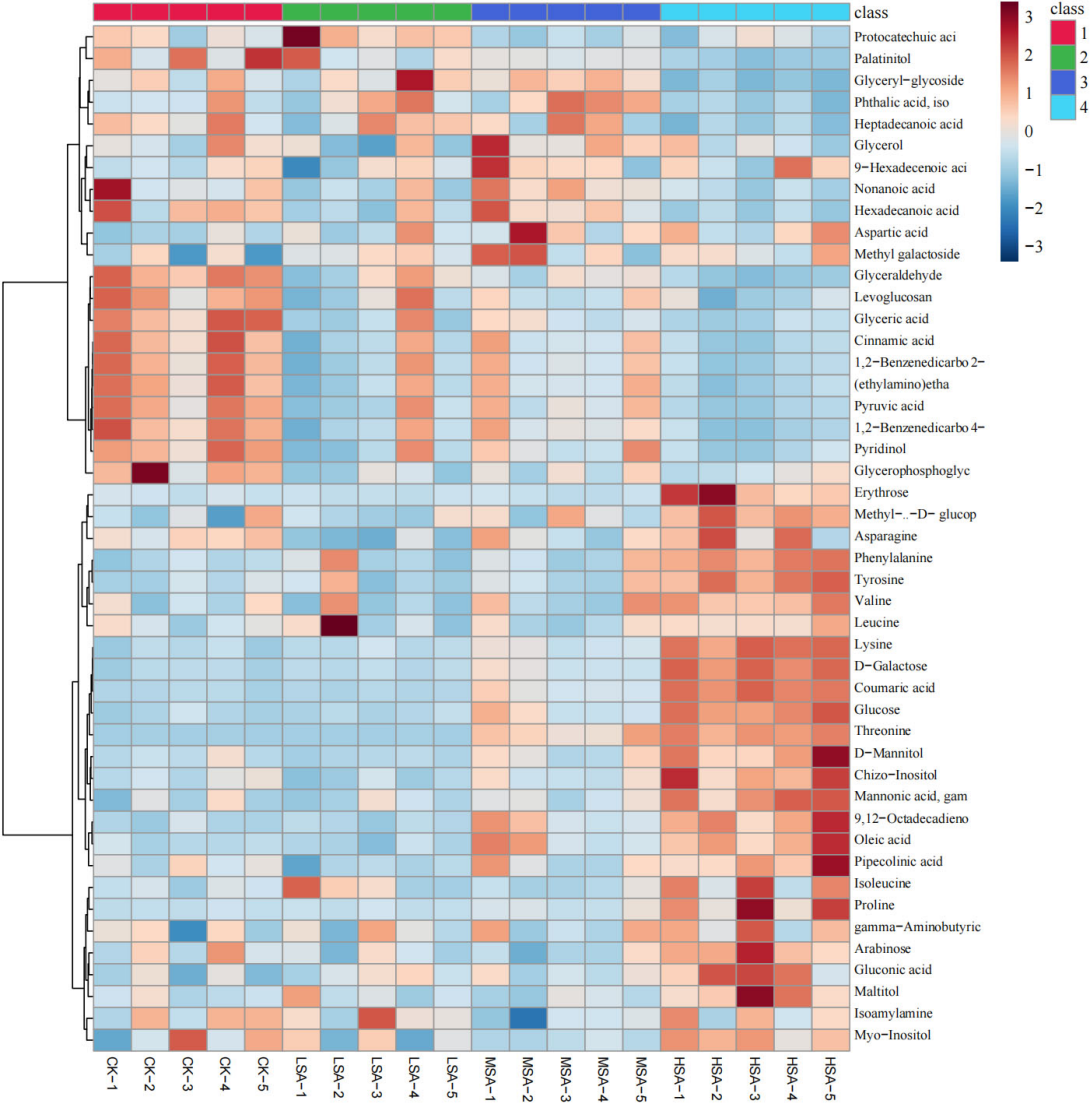
The clustering heat map of characteristic metabolites in tobacco-planting soil treated with different concentrations of succinic acid (VIP > 1.2 and *p* < 0.05, CK, LSA, MSA, and HSA). Red indicates upregulation, whereas blue indicates downregulation.

To obtain characteristic metabolites, pairwise comparison by PLS-DA was used to screen potential biomarkers with VIP > 1.2 and *p* < 0.05. The clustering heat map revealed the changing trends of screened 47 characteristic metabolites in tobacco-planting soil (Figure 4). Compared with the control group, 0.4% succinic acid had the most obvious regulatory effect on soil metabolites and increased the contents of four sugar alcohols - myo-inositol, maltitol, chizo-inositol and D-mannitol, as well as two sugar acids - gluconic acid and mannonic acid, five monosaccharides - arabinose, glucose D-galactose, methy-α-D-glucopyranoside and erythrose, eleven amino acids - gama-aminobutyric acid, proline, isoleucine, pipecolinic acid, threonine, lysine, leucine, valine, tyrosine, phenylalanine and asparagine, two long-chain fatty acid - 9,12-octadecadienoic acid (*Z, Z*) and oleic acid. Meanwhile, the application of 0.4% succinic acid decreased the contents of glyceraldehyde, levoglucosan, glyceric acid, coumaric acid, 2-(ethylamino)ethanol, pyruvic acid, 4-pyridinol and glycerophosphoglycerol in the soil. Although the concentrations of succinic acid added in the soil were different, the difference of succinic acid among different treatment groups were relatively small due to the absorption and utilization by microbes.

**FIGURE 5:**
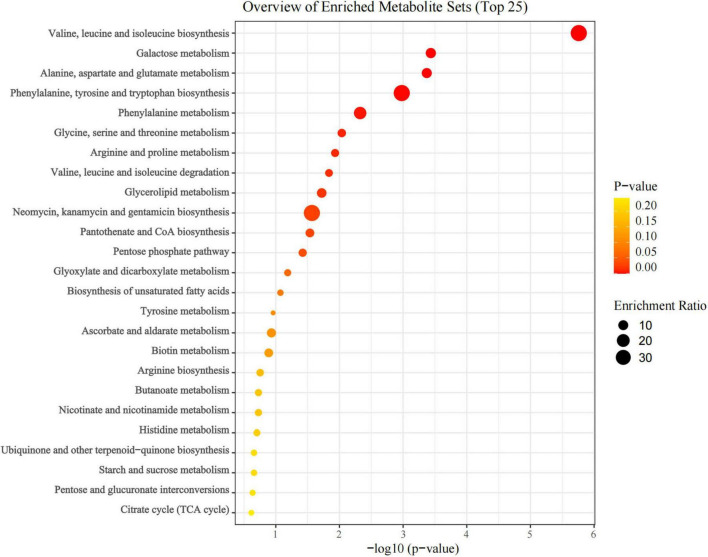
Enrichment bubble diagram of metabolic pathways of characteristic metabolites in tobacco-planting soil treated with different concentrations of succinic acid (CK, LSA, MSA, and HSA). Large sizes and red colors represent the major pathway enrichment and high pathway impact values, respectively.

The top 25 differential metabolic pathways were identified with enrichment analysis with 47 characteristic metabolites, as shown in Figure 5. They were significantly enriched in both amino acid metabolism and carbohydrate metabolism. The first five metabolic pathways are valine, leucine and isoleucine biosynthesis, galactose metabolism, alanine, aspartate and glutamate metabolism, phenylalanine, tyrosine and tryptophan biosynthesis and phenylalanine metabolism. At the same time, significant metabolic pathways also include glycine, serine and threonine metabolism, arginine and proline metabolism, valine, leucine and isoleucine degradation, glycerolipid metabolism, neomycin, kanamycin and gentamicin biosynthesis.

### 3.4 Correlation between microbes and soil metabolites

**FIGURE 6:**
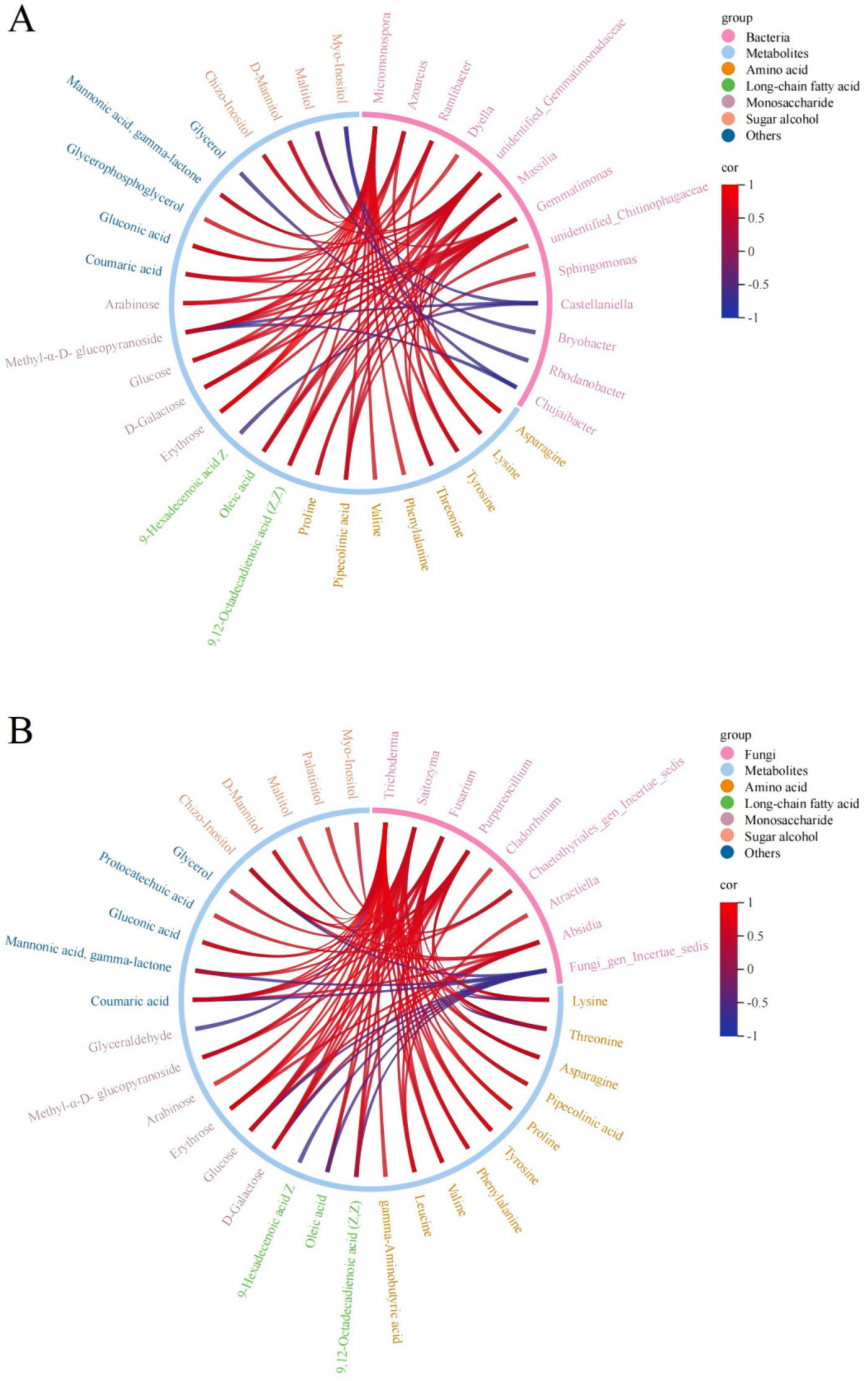
The chord diagrams between differential bacterial communities **(A)** and fungi communities **(B)** at the genus level and characteristic metabolites. The red lines represent strong positive correlations (r > 0.6, *p* < 0.05), and the blue lines represent strong negative correlations (r < −0.6, *p* < 0.05).

The characteristic metabolites which have significant correlations with microbes are mainly amino acids and sugar substances (Figure 6). The correlation analysis between differential bacterial communities at the genus level and characteristic metabolites indicated that *Micromonospora*, unidentified *Gemmatimonadaceae*, and *Gemmatimonas* had more diverse associations with characteristic metabolites and were positively correlated. Bacterial genera such as *Azoarcus*, *Ramlibacter*, and *Massilia* were also positively correlated with related metabolites. Compared with other types of metabolites, amino acids were positively correlated with bacterial genera. The correlation analysis between differential fungal communities at the genus level and characteristic metabolites indicated that *Trichoderma*, *Saitozyma*, and *Purpureocillium* were all positively correlated with the characteristic metabolites. Compared with the bacterial community, there were more characteristic metabolites significantly correlated to the fungal community and the molecular networks were more complex.

### 3.5 Tobacco chemical components

**FIGURE 7:**
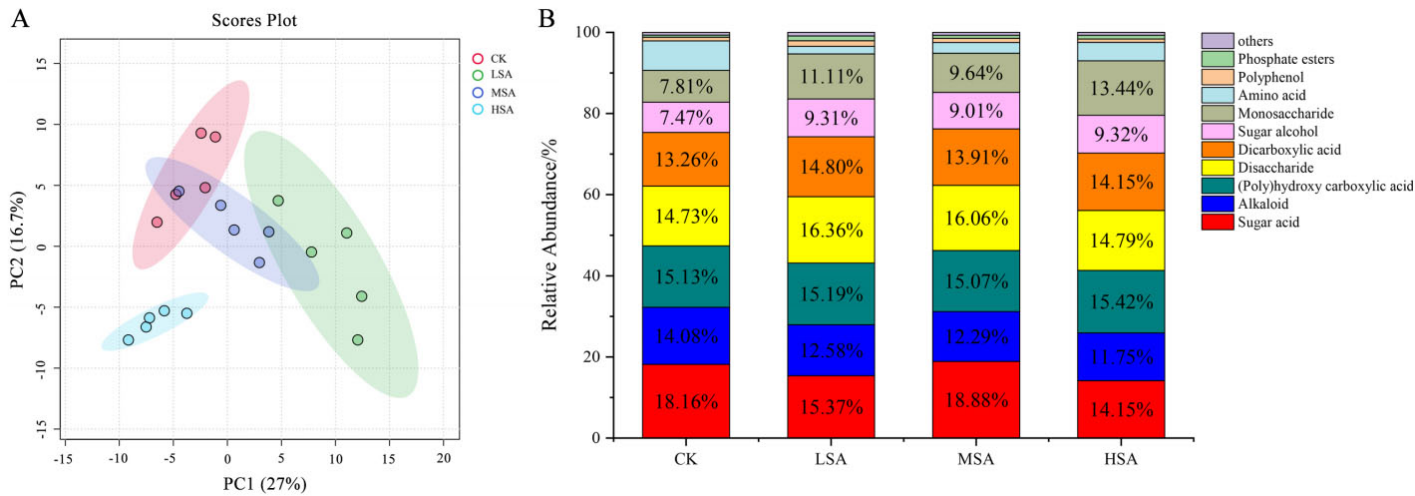
The principal component analysis **(A)** and the top 10 and other chemical classifications relative abundance **(B)** of tobacco leaf metabolites treated with different concentrations of succinic acid (CK, LSA, MSA, and HSA).

After addition of different concentrations of succinic acid to the tobacco-planting soil, the chemical components of tobacco leaf changed significantly, and LSA, MSA, and HSA were significantly different from the control group (Figure 7A). In the bar chart of the relative abundance of tobacco chemical components, the addition of succinic acid increased the relative abundance of disaccharide, sugar alcohol and monosaccharide and decreased the relative abundance of alkaloid and amino acid, playing a certain regulatory role in the tobacco chemical components (Figure 7B).

## 4 Discussion

### 4.1 Response of soil chemical properties to succinic acid

A total of 0.4% succinic acid regulated the soil chemical properties and had a positive effect on soil fertility. The input of succinic acid as an external carbon source disrupted the carbon-nitrogen ratio balance, thereby maybe affect the transformation rate of humus in soil organic matter and the rate of soil mineralization (Tian et al., 2017). When the carbon-nitrogen ratio in the soil is too high, microbes need to consume more available nitrogen to maintain normal physiological activities, which results in an increase in the release of total nitrogen and hydrolyzed nitrogen (Kuypers et al., 2018). At the same time, the input of exogenous organic carbon can increase the content of soil available phosphorus and available potassium, meet the needs of crop growth and improve crop yield (Hadi et al., 2021). The decrease of soil pH may be caused by the change in the ratio of anions and cations after succinic acid application. Although the pH decreased, it still remained within the optimal range of 5.5–6.5 for tobacco cultivation (Feng et al., 2023). After succinic acid application, AK, TN and TK content showed a significant change compared with CK. As a driving factor of other chemical properties, pH significantly affects the transformation and accumulation of nitrogen, phosphorus and potassium elements in the soil, while the changes in the availability of N, P, and K also affect the soil microbial community and plant growth (Cerozi and Fitzsimmons, 2016).

### 4.2 Response of soil microbes to succinic acid

The addition of small molecule metabolites to the soil can promote or inhibit the reproduction of some microbial communities, and then result in forming new microbial community structures, thereby changing the richness and diversity of microbial communities (Debode et al., 2016). As one of soil organic acid metabolites, succinic acid can attract the chemotaxis and colonization of certain bacteria and fungi, and attract beneficial bacteria to colonize in the rhizosphere region (Tan et al., 2013), which may be one of the reasons for the decrease of bacterial OTUs and diversity index. The imbalance of the carbon-nitrogen ratio may also lead to a decreasing trend in the OTUs and diversity index of bacteria and fungi (Kieloaho et al., 2016). In addition, the application of succinic acid alters the soil chemical properties as pH, AK, TN and TK. These soil chemical indicators reconstruct soil metabolites, ultimately interacting with the soil microbial community (Wang et al., 2022).

The addition of succinic acid led to soil microbes competing with each other for resource substances and ecological sites required for colonization. The negative interactions among species in the soil microbial community increase, thereby regulating the soil microbial community (Li et al., 2019). In the bacterial community, *Proteobacteria*, *Actinobacteriota*, *Chloroflexi*, and *Gemmatimonadota* are the dominant bacterial phyla. *Proteobacteria is* the main user of plant root secretions, which can positively respond to low molecular weight substances (Tian et al., 2019). The addition of high concentration succinic acid increased the abundance of nitrogen-fixing bacteria such as *Rhizobiales*, *Sphingomonadaceae*, and *Sphingomonas* in the *Proteobacteria* phylum. Their abundance is usually positively correlated with the carbon-nitrogen ratio, carbon concentration and nitrogen concentration (Li et al., 2025). The enrichment of nitrogen-fixing bacteria in the soil environment can promote biological nitrogen fixation and the production of plant hormones to enhance the adaptability of plants (Wang et al., 2012). *Chloroflexi* is metabolically diverse and has a high degradation capacity. Therefore, it has a significant advantage when competing with other microbes for nutrients and living space (Rao et al., 2022), and thus its abundance increases significantly at addition of succinic acid. Among the biomarkers identified by linear discriminant analysis effect size, *Micromonosporaceae* and *Micromonosporales*, which can synthesize special metabolites to promote plant growth and recruit plant probiotics, are key biomarkers in HSA (Carro et al., 2018).

In the fungal community, *Ascomycota*, *Chytridiomycota*, and *Basidiomycota* are the main dominant phyla. In this study, the relative abundance of *Ascomycota* and *Basidiomycota* has increased. Most *Ascomycota* are saprophytic and can decompose chitin and lignin, and *Basidiomycota* are also decomposers of lignin and cellulose and can form beneficial mycorrhizal associations with plants (Xiong et al., 2014). The addition of high concentration succinic acid increased the content of organic matter and activated carbon in the soil, significantly enhancing their relative abundance (Wang et al., 2021). In addition, *Ascomycota* have richer species diversity and a faster evolutionary rate compared with other fungal communities. Therefore, they can quickly strive for favorable conditions to promote their own reproduction (Lutzoni et al., 2004). The decrease in soil pH caused by succinic acid not only alters the ecological niche distribution of microbes, but also directly affects the ion exchange capacity and nutrient availability in the soil (Liu et al., 2018). This resulting change in the competitive relationship among communities and the efficiency of resource utilization may have imposed physiological limitations on the growth of some fungi, thereby leading to a decrease in the abundance of *Chytridiomycota* (Wang et al., 2024). At lower taxonomic levels, *Hypocreales* and *Trichoderma* are biomarkers of HSA. The *Hypocreales* and *Trichoderma* can inhibit plant pathogenic bacteria, promote the healthy growth of plants, and increase crop yields (Jiang et al., 2024; Soltani and Moghaddam, 2015).

Soil bacterial function prediction indicates that significant changes have occurred in some bacterial groups related to the C and N cycles in HSA (such as aerobic chemoheterotrophy, chemoheterotrophy, nitrate reduction, nitrogen respiration, nitrate respiration, etc.), suggesting that the addition of high concentration succinic acid might affect the nutrients and carbon-nitrogen cycles. Chemoheterotrophy and aerobic chemoheterotrophy bacteria are often used as the main energy source for the food web of soil microbial communities (Dong et al., 2020). They obtain carbon and energy by oxidizing a large amount of succinic acid, forming a relatively high nutrient conversion efficiency, and promoting nutrient absorption and nutrient cycling in the root system (Wu et al., 2021). Among them, the relative abundances such as nitrate reduction, nitrogen respiration, and nitrate respiration were the highest in HSA. This might be because high concentration of succinic acid significantly increased key environmental factors such as soil organic matter and total nitrogen, increased the microbial abundance related to the nitrogen cycle (Arrobas et al., 2025).

Saprophytic fungi are important participants in the nutrient cycle between soil and litter, and are closely related to the carbon cycle and organic matter decomposition. In fungal function prediction, the significant increase in undefined saprotroph might be related to the elevated carbon input. The addition of high concentration of succinic acid led to an increase in available nutrients in soil, promoting the growth of saprophytic fungi (Wei et al., 2020). Compared with the control group, the addition of succinic acid led to a decrease in the abundance of ectomycorrhizal. The abundance of ectomycorrhizal fungi was positively correlated with *Basidiomycota*, because *Basidiomycota* can form ectomycorrhizal with plant roots and assist plants in obtaining mineral nutrients under carbon-limited conditions (Basiru et al., 2025). Bacteria tend to give priority to using easily degradable carbon sources, while fungi utilize complex compounds. Meanwhile, bacteria also compete with fungi for the products released during the degradation process of complex substrates. Therefore, the different responses of bacterial and fungal communities structure and function to changes in succinic acid input may be related to their differences in resource utilization (Wang and Kuzyakov, 2024).

### 4.3 Response of soil metabolites to succinic acid

Through the GC-MS environmental pseudotargeted metabolomics analysis, it was found that the composition and structure of soil metabolites significant changed, due to the addition of exogenous succinic acid, as a medium for the co-evolution of plants and microbes. Among the types of compounds exuded from plant roots, sugars are usually regarded as the compounds with the highest total exudation richness (Moe, 2013). These sugars can not only be consumed as an energy and carbon source for soil animals, plants and microbes, but also be re-synthesized by microbes and released into the soil. In this study, the absolute contents of monosaccharide, disaccharide, sugar alcohol and trehalose have significantly increased. As a soil carbohydrate, succinic acid can affect soil chemical processes, plant nutrition and microbial activity, and thereby influencing plant root function and crop growth, and thus regulating the distribution of sugar. The change in sugar content is mainly related to the biomass of the fungal community or nutritional status, while the addition of succinic acid reconstructed the soil microbial community and thereby affects the consumption and release of sugar substances (Medeiros et al., 2006).

The regulatory effect of 0.4% succinic acid on soil characteristic metabolites is the most obvious, especially amino acid. In HSA, eleven amino acids such as gama-aminobutyric acid, proline, and isoleucine are significantly upregulated. The level and distribution of amino acids are not only controlled by plants, but also related to the functions of microbes, and can serve as a nitrogen source for plants and microbes in a nitrogen-restricted environment (Wang et al., 2011). Soil microbes can easily chemically transfer amino acids, and the presence of amino acids contributes to the richness of the microbial community at the plant root-soil interface. The abundance of *Rhizobiales*, *Sphingomonadaceae*, and *Sphingomonas* has increased in this study. Therefore, changes in amino acid content may directionally regulate these nitrogen-fixing bacteria and have the function of recruiting beneficial microbes to the rhizosphere (Pratelli et al., 2010). The content of sugar alcohols was also significantly upregulated in the soil, mainly manifested in myo-inositol, maltitol, chizo-inositol and D-mannitol. Sugar alcohol can promote the growth of plants, bacteria, fungi and yeasts under environmental stress. For instance, mannitol can act as a soil conditioner to influence the functional diversity of soil microbial communities and enzyme activities, thereby altering soil fertility and quality (Yu et al., 2016). The addition of exogenous succinic acid not only affected the production of various secondary metabolites, but also regulated the basic microbial metabolic processes. In the KEGG pathway enrichment analysis, the characteristic metabolites are mainly enriched in amino acid metabolism and carbohydrate metabolism. Amino acids are the main energy source for microbial metabolism and also the sources of synthesis for various macromolecular proteins with biological functions (López-González et al., 2015). The changes in amino acid metabolism help microbes absorb amino acids and promote the utilization of nitrogen by plants. Carbohydrate, like sugar, is the energy source for microbial metabolism. The growth of plants and the reproduction of microbes are closely related to the abundance of soil carbohydrate transport and metabolism (Shi-yu Zhou et al., 2019). Therefore, carbohydrate metabolism is significantly enriched in HSA treatment.

### 4.4 Interaction between microbial communities and metabolites and its influence on tobacco chemical components

The changes of the ecological environment, agricultural management and the addition of exogenous organic matter can all alter the interaction between soil microbes and metabolites. Key microbial groups and characteristic metabolites play unique and crucial roles, and their changes will greatly affect the structure, function and correlation of the microbiome and metabolome (Banerjee et al., 2018). This study found that the differential bacteria at the genus level were mostly positively correlated with the characteristic metabolites. *Micromonospora* can not only promote plant growth and inhibit pathogens by synthesizing antifungal and antibacterial compounds, but also produce various hydrolases to decompose plant cell wall (Hirsch and Valdés, 2010). This might be the reason why *Micromonospora* is positively correlated with most of metabolites. C and N are the most important resources for bacterial growth. The addition of succinic acid will significantly enrich specific microbial groups that are highly associated with soil C and N. *Gemmatimonas* is positively correlated with soil organic carbon and total nitrogen, and can regulate the intake of C and N according to their metabolic requirements under various conditions (Li et al., 2017). Therefore, it is positively correlated with soil metabolites. Bacterial communities at the genus level such as *Azoarcus*, *Ramlibacter*, *Ramlibacter* also show a significant positive correlation with metabolites.

One bacterial and four fungal biomarkers had a strong correlation with characteristic metabolites, indicating that soil metabolites are mainly affected by fungi, and the response of the fungal community to the application of exogenous succinic acid is greater than that of the bacterial community. *Trichoderma*, *Saitozyma*, *Purpureocillium*, and *Absidia* were positively correlated with a variety of metabolites, including amino acids, monosaccharides and sugar alcohols, suggesting that these metabolites may originate from fungi (Tan et al., 2023). Studies have shown that fungi have a higher growth efficiency than bacteria. They can convert more carbohydrates into complex and stable microbial source carbon and emit less CO_2_ (Bailey et al., 2002). Therefore, abundant fungi can retain carbohydrates more effectively, which might be reason why fungi have a stronger relationship with metabolites.

Plants respond to various abiotic stresses by changing in primary metabolism. The addition of succinic acid induced comprehensive alterations in soil chemical properties, metabolic profiles, and microbial communities, consequently triggering metabolic pathway change in tobacco leaves and regulating leaf chemical composition. Plant pseudotargeted metabolomics analysis revealed that succinic acid application significantly altered the tobacco chemical composition. The contents and proportions of various chemical components have been improved mainly including sugar acid, alkaloid, (poly)hydroxy carboxylic acid, disaccharide, sugar alcohol and monosaccharide. The sweetness and mellowness of tobacco leaf aroma are determined by water-soluble sugars. Within a certain range, the increase in water-soluble total sugar content is positively correlated with the improvement of tobacco leaf quality (Zhou et al., 2017). The decreasing alkaloid levels in tobacco can potentially enhance the perception of aroma quality and quantity (Jiang et al., 2017). An optimal sugar/alkaloid ratio of 6–10, with 10 being the best (Cai et al., 2023a). The addition of succinic acid to a certain extent reduces the relative abundance of alkaloid and increases the relative abundance of disaccharide, sugar alcohol and monosaccharide, indicating that succinic acid can coordinate the tobacco chemical composition.

## 5 Conclusion

In this study, considering beneficial metabolites as potential prebiotics, we focused on the effects of different concentrations of succinic acid on the microbial community and metabolite composition in soil, as well as the interaction between them. 0.4% succinic acid can effectively regulate the soil chemical properties and has a positive impact on soil fertility. Compared with the bacteria, succinic acid had a more significant impact on the structure of fungal communities. The microbial functional prediction showed 0.4% succinic acid might enhance soil nutrients and carbon-nitrogen cycle. The absolute contents of monosaccharide, disaccharide, sugar alcohol and trehalose in soil metabolites increased significantly and the screened 47 characteristic metabolites were mainly enriched in amino acid metabolism and carbohydrate metabolism. The correlation between microbes at the genus level and characteristic metabolites of sugar and amino acid was closer and mainly positive. These interaction increased the relative abundance of monosaccharide, disaccharide and sugar alcohol and decrease alkaloid and amino acid in tobacco leaves, coordinated the tobacco chemical composition. However, the economic and environmental benefits of long-term succinic acid application still require continuous evaluation, particularly in terms of its field application rate and effects on soil pH.

## Data Availability

The data presented in the study are deposited in the NCBI repository, accession numbers PRJNA1188346 and PRJNA1188357.

## Footnotes

1
https://new.metaboanalyst.ca/
