# Supplementary material for: Succinic acid improves crop chemical components by regulating soil chemical properties, microbes and metabolites
Source: Front Microbiol. 2025 Oct 21;16:1674309. doi: 10.3389/fmicb.2025.1674309 (PMC12583033; doi:10.3389/fmicb.2025.1674309)

**Supplementary material**

**Succinic Acid Improves Crop Chemical Components by Regulating Soil Chemical Properties, Microbes and Metabolites**

**Figures**

**Figure S1** Venn graph of the bacterial (**A**) and fungal (**B**) OTUs (operational taxonomic units) identified in the tobacco planting soil.


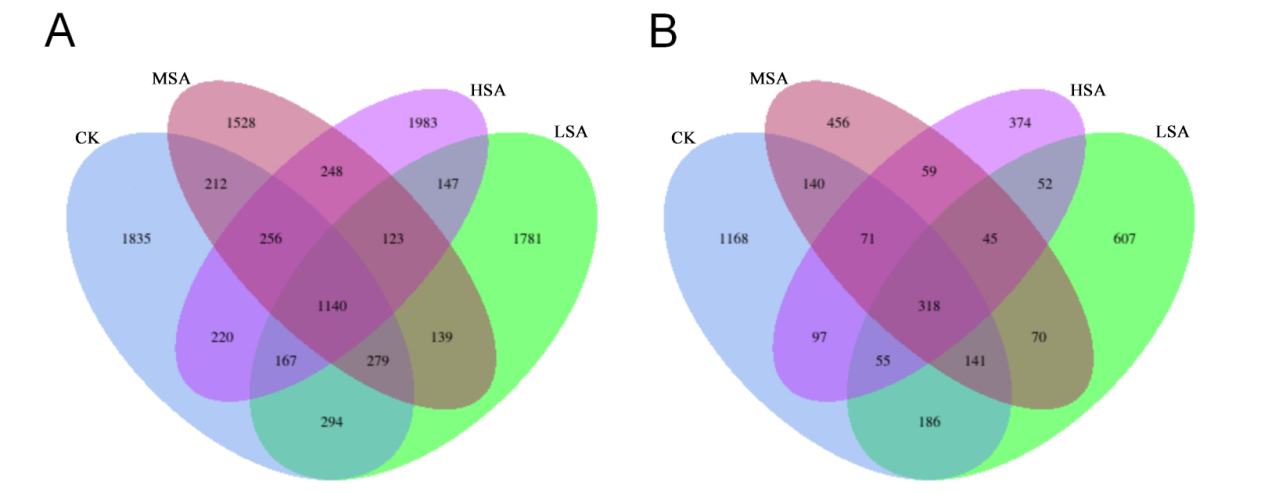


**Figure S2** Box plot of differences between groups of the bacterial (**A**, **B**, **C**) and fungal (**D**, **E**, **F**) Alpha diversity index in the tobacco planting soil.

**
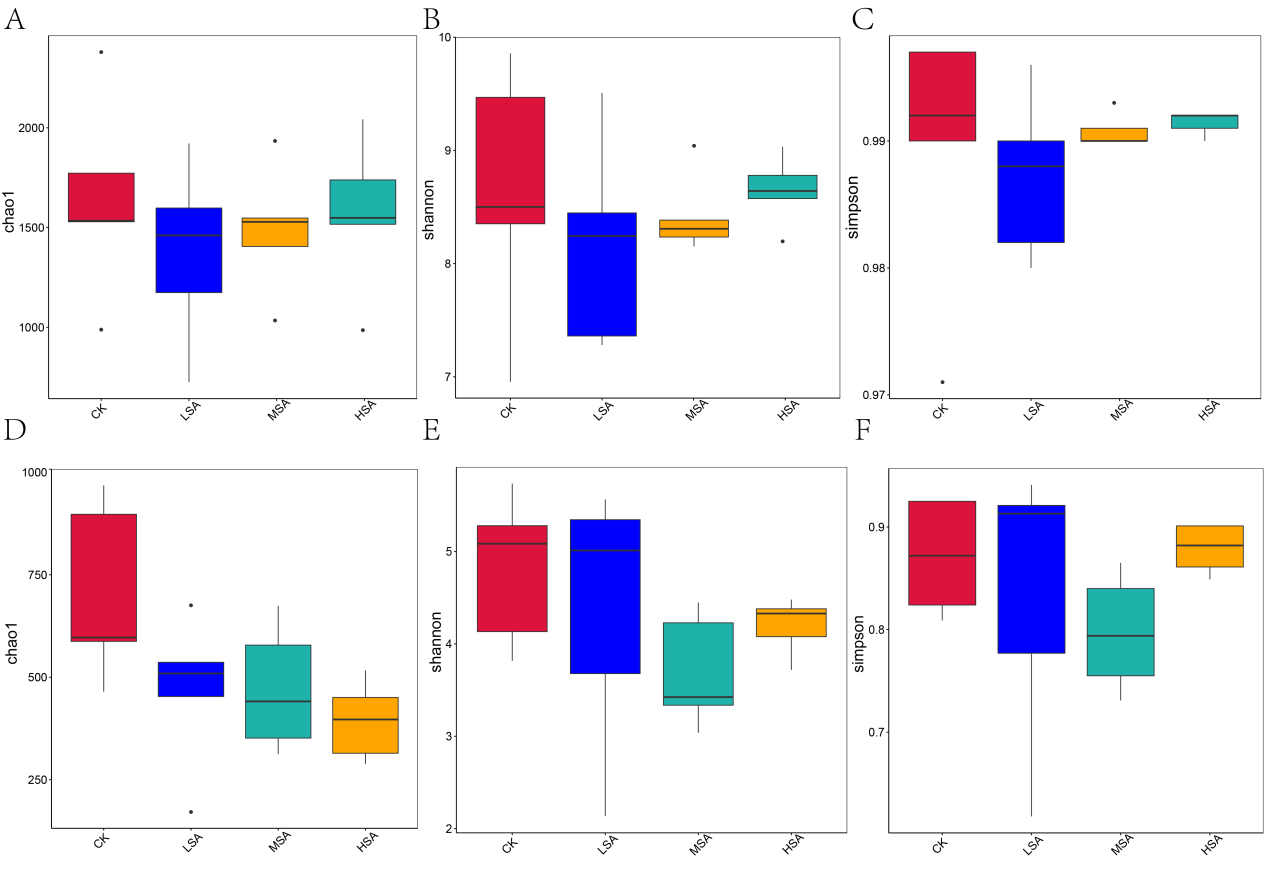
**

**Figure S3** Abundance clustering heat map of bacteria (**A**) and fungi (**B**) in tobacco-planting soil at the genus level.


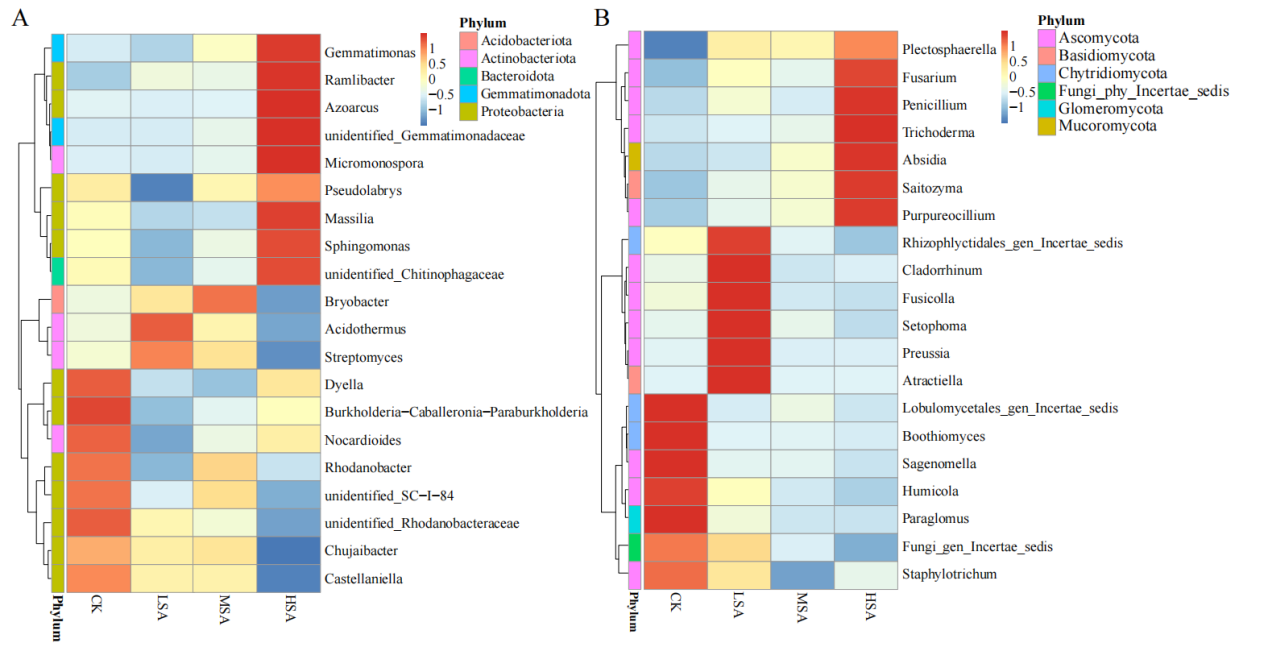


**Figure S4** Clustering heat map of functional abundance of bacteria (**A**) and fungi (**B**) in tobacco-planting soil with the FAPROTAX and FUNGUILD databases.


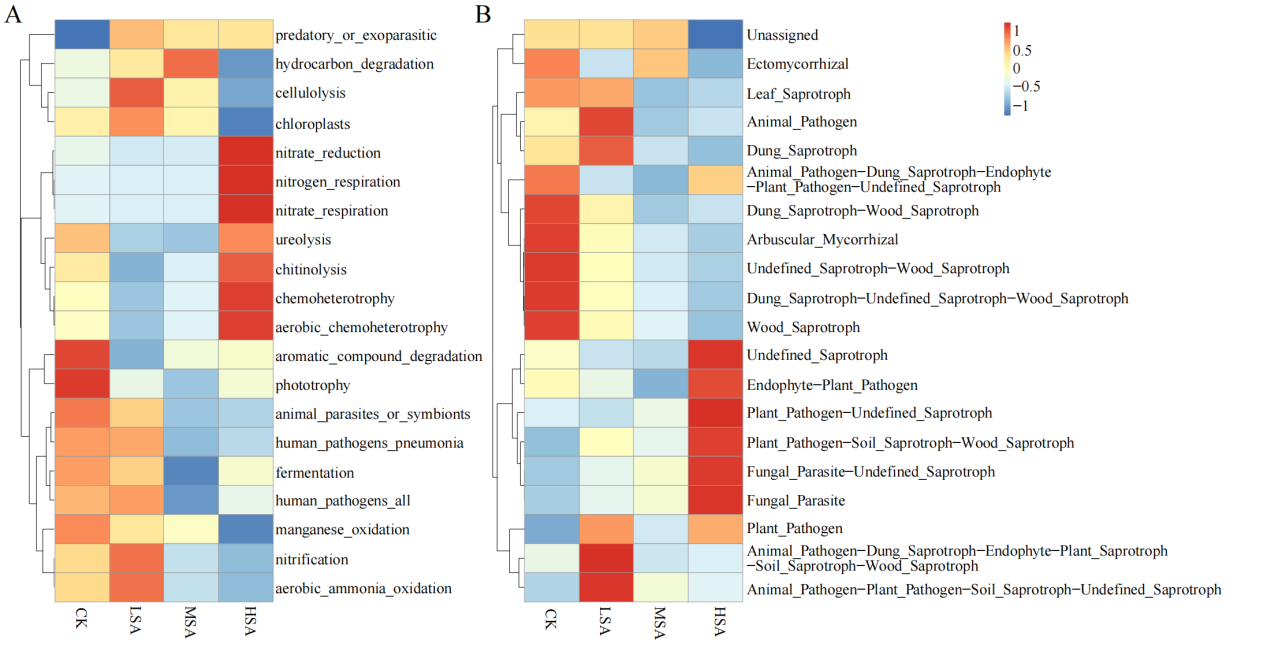

Supplement: Supplementary file 1 [file Data_Sheet_1.docx]
